# Diagnostic Performance of Radial EBUS‐Guided Cryobiopsy for Peripheral Lung Lesions: First Experience in Morocco

**DOI:** 10.1111/crj.70216

**Published:** 2026-07-23

**Authors:** Lamya Chrif Morand, Khalid Bouti, Imane Saidi, Soumia Fdil, Anis Rafik

**Affiliations:** ^1^ Interventional Pulmonology Division International Hospital Kenitra Akdital Kenitra Morocco; ^2^ Department of Pulmonology Mohammed VI General University Hospital Tangier Morocco; ^3^ Laboratory of Life and Health Sciences, Faculty of Medicine and Pharmacy of Tangier Abdelmalek Essaâdi University Tétouan Morocco; ^4^ Faculty of Medical Sciences, UM6P Hospitals University Mohammed VI Polytechnic Benguerir Morocco; ^5^ Department of Respiratory Medicine Military Mohamed V Hospital Rabat Morocco

**Keywords:** bronchoscopy, cryobiopsy, endobronchial ultrasound, interventional pulmonology, peripheral pulmonary lesions, transbronchial biopsy

## Abstract

**Introduction:**

Accurate diagnosis of peripheral pulmonary lesions (PPLs) remains challenging. Conventional forceps biopsy frequently yields small samples compromised by crush artifact. Transbronchial cryobiopsy (TBCB) offers larger, better‐preserved specimens. This study evaluated the diagnostic yield and safety of radial endobronchial ultrasound (r‐EBUS)‐guided TBCB using a 1.1‐mm cryoprobe.

**Materials and Methods:**

This prospective single‐center study enrolled 73 consecutive patients with PPLs. Procedures were performed under general anesthesia using a laryngeal mask airway, a flexible bronchoscope, r‐EBUS for localization, and a 1.1‐mm cryoprobe inserted through a guide sheath. A prophylactic bronchial balloon blocker was employed for hemorrhage control. The primary outcome was diagnostic yield; secondary outcomes included bleeding severity and pneumothorax incidence.

**Results:**

Mean lesion diameter was 21 mm, with 79.5% of patients exhibiting a positive bronchus sign. A definitive histological diagnosis was established in 55 of 73 patients (75.3%). Malignancies accounted for 60.3% of diagnoses, whereas benign etiologies, including tuberculosis, represented 15.1%. Bleeding occurred in 19.2% of cases; all events were mild and managed with the balloon blocker. No severe hemorrhage was observed. Pneumothorax occurred in six patients (8.2%), with four requiring chest tube drainage.

**Conclusion:**

r‐EBUS‐guided TBCB using a 1.1‐mm cryoprobe is feasible and effective for diagnosing PPLs, with a diagnostic yield of 75.3%. Protocolized use of a balloon blocker effectively prevents severe hemorrhagic complications. Pneumothorax risk warrants vigilance, particularly in patients with infectious cavitary disease.

## Graphical abstract

Radial EBUS‐guided transbronchial cryobiopsy using a 1.1‐mm cryoprobe achieved a 75.3% diagnostic yield for peripheral lung lesions in 73 patients, with no severe hemorrhage. Protocolized balloon blocker use effectively controlled all bleeding events, supporting cryobiopsy as a feasible, safe alternative to conventional forceps biopsy.

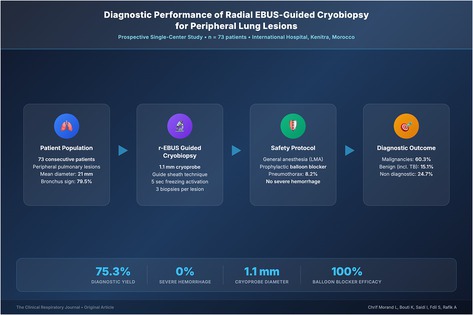

## 1 Introduction

The accurate diagnosis of peripheral pulmonary lesions (PPLs) remains a significant challenge in interventional pulmonology. Although the advent of radial endobronchial ultrasound (r‐EBUS) has standardized lesion localization, conventional sampling methods such as forceps biopsy often provide small specimens with crush artifact, limiting the ability to perform necessary molecular analysis and histological subtyping [1, 2]. Consequently, there is a growing clinical need for techniques that maximize tissue acquisition without compromising safety [3, 4].

Transbronchial cryobiopsy (TBCB) has emerged as a superior alternative to mechanical biopsy, capable of retrieving significantly larger tissue samples with preserved architecture [5, 6]. Although initially popularized for interstitial lung disease, its application for PPLs is increasing. Recent randomized trials and prospective studies suggest that thinner 1.1‐mm cryoprobes can effectively navigate to distal airways, achieving diagnostic yields comparable to larger probes while potentially offering better maneuverability [7, 8].

Despite these advantages, concerns regarding procedural safety, specifically the risks of severe hemorrhage and pneumothorax, persist in the literature [7]. Furthermore, the optimal integration of cryobiopsy with specific guidance protocols requires ongoing validation in diverse clinical settings. The primary objective of this prospective observational study was to evaluate the diagnostic yield and safety profile of r‐EBUS‐guided TBCB utilizing a 1.1‐mm cryoprobe for the diagnosis of PPLs.

## 2 Materials and Methods

### 2.1 Study Design and Ethics

This prospective observational study was conducted at the International Hospital in Kenitra. The primary objective was to evaluate the diagnostic yield and safety profile of TBCB for the sampling of PPLs. The study protocol was approved by the institutional review board, and written informed consent was obtained from all participants prior to the procedure.

### 2.2 Participants

The study population comprised 73 consecutive patients presenting with PPLs requiring histological diagnosis. Baseline demographic data, including age and sex, were recorded. Lesion characteristics, specifically size (diameter), lobar location, and the presence or absence of a bronchus sign, were assessed prior to the intervention.

### 2.3 Procedures

All bronchoscopic procedures were performed under general anesthesia utilizing a laryngeal mask airway to ensure stable airway control. Patient selection followed an all‐comers approach, with every consecutive patient referred for histological characterization of a PPL enrolled regardless of lesion size or bronchus sign status. Lesion localization was primarily achieved using an Olympus r‐EBUS probe. The “Bronchus Archimède” laptop‐based virtual navigation system was used to facilitate pathway planning in a subset of patients. No formal selection criteria for virtual navigation were pre‐specified in the study protocol; its use was left to the operator's discretion, who tended to favor navigation in lesions perceived as more challenging to reach, typically smaller nodules, lesions without a bronchus sign on pre‐procedural CT, and lesions located in anatomically difficult segments such as the upper lobes.

The procedural setup involved a flexible bronchoscope with a 1.7‐mm working channel used in conjunction with a guide sheath. Once the target lesion was visualized via r‐EBUS and confirmed with fluoroscopy, the radial probe was withdrawn, and an Erbe 1.1‐mm cryoprobe was inserted through the guide sheath. Tissue sampling was performed with a standardized freezing activation time of 5 s. The protocol mandated the acquisition of three biopsies per nodule to maximize diagnostic potential.

To ensure procedural safety, a prophylactic bronchial balloon blocker was utilized for bleeding prevention and management. Continuous fluoroscopic monitoring was maintained throughout the procedure to verify instrument position and assess for immediate complications.

### 2.4 Outcomes and Definitions

The primary outcome measure was diagnostic yield, defined as the percentage of cases in which a definitive histological diagnosis was established. Secondary outcomes focused on procedural safety, specifically the incidence and severity of bleeding and pneumothorax. Bleeding severity was stratified into mild or severe categories based on the volume of blood loss and the necessity for hemostatic intervention. Pneumothorax cases were recorded and categorized based on the management strategy required, distinguishing between those managed conservatively and those necessitating chest tube drainage.

### 2.5 Statistical Analysis

Data were analyzed using descriptive statistics. Continuous variables, such as patient age and lesion diameter, were expressed as means with ranges. Categorical variables, including diagnostic yield, histological subtypes, and complication rates, were reported as frequencies and percentages.

## 3 Results

### 3.1 Baseline Characteristics and Study Population

A total of 73 consecutive patients presenting with PPLs were enrolled in this prospective observational study. All participants underwent TBCB under general anesthesia following the established protocol. The study population exhibited a male predominance, with 47 male patients (64.4%) and 26 female patients (35.6%). The mean age of the cohort was 64 years, with a range spanning from 37 to 79 years.

Pre‐procedural assessment of lesion characteristics was performed for all subjects. The mean diameter of the target peripheral lesions was 21 mm (range: 12–30 mm). Stratification by size revealed a relatively balanced distribution: 33 lesions (45.2%) were classified as small nodules (less than 20 mm), whereas 40 lesions (54.8%) measured greater than 20 mm. Regarding anatomical distribution, the majority of lesions were located in the upper lobes (*n* = 45; 61.6%), with the remaining 28 lesions (38.4%) situated in the middle or lower lobes.

A crucial factor for bronchoscopic accessibility, the computed tomography (CT) bronchus sign, was evaluated prior to the procedure. A positive bronchus sign, indicating the presence of a bronchus leading directly to or contained within the lesion, was identified in 58 patients (79.5%). Conversely, 15 patients (20.5%) presented with lesions lacking a definitive bronchus sign. The baseline demographics and lesion characteristics are summarized in Table 1.

**TABLE 1:** Baseline demographic and clinical characteristics of the study population.

Characteristic	Value (*N* = 73)
Age (years)	
Mean	64
Range	37–79
Sex, *n* (%)	
Male	47 (64.4%)
Female	26 (35.6%)
Lesion diameter	
Mean (range)	21 mm (12–30 mm)
≤ 20 mm	33 (45.2%)
> 20 mm	40 (54.8%)
Location, *n* (%)	
Upper lobes	45 (61.6%)
Other lobes	28 (38.4%)
Bronchus sign, *n* (%)	
Positive	58 (79.5%)
Negative	15 (20.5%)

### 3.2 Diagnostic Yield

The primary outcome of the study was the overall diagnostic yield, defined as the proportion of patients for whom a definitive histological diagnosis was successfully established via TBCB. A definitive diagnosis was obtained in 55 of the 73 patients, resulting in an overall diagnostic yield of 75.3%. In the remaining 18 patients (24.7%), the biopsy specimens were non‐diagnostic, showing either nonspecific benign tissue or insufficient material for a conclusive pathological determination.

**FIGURE 1:**
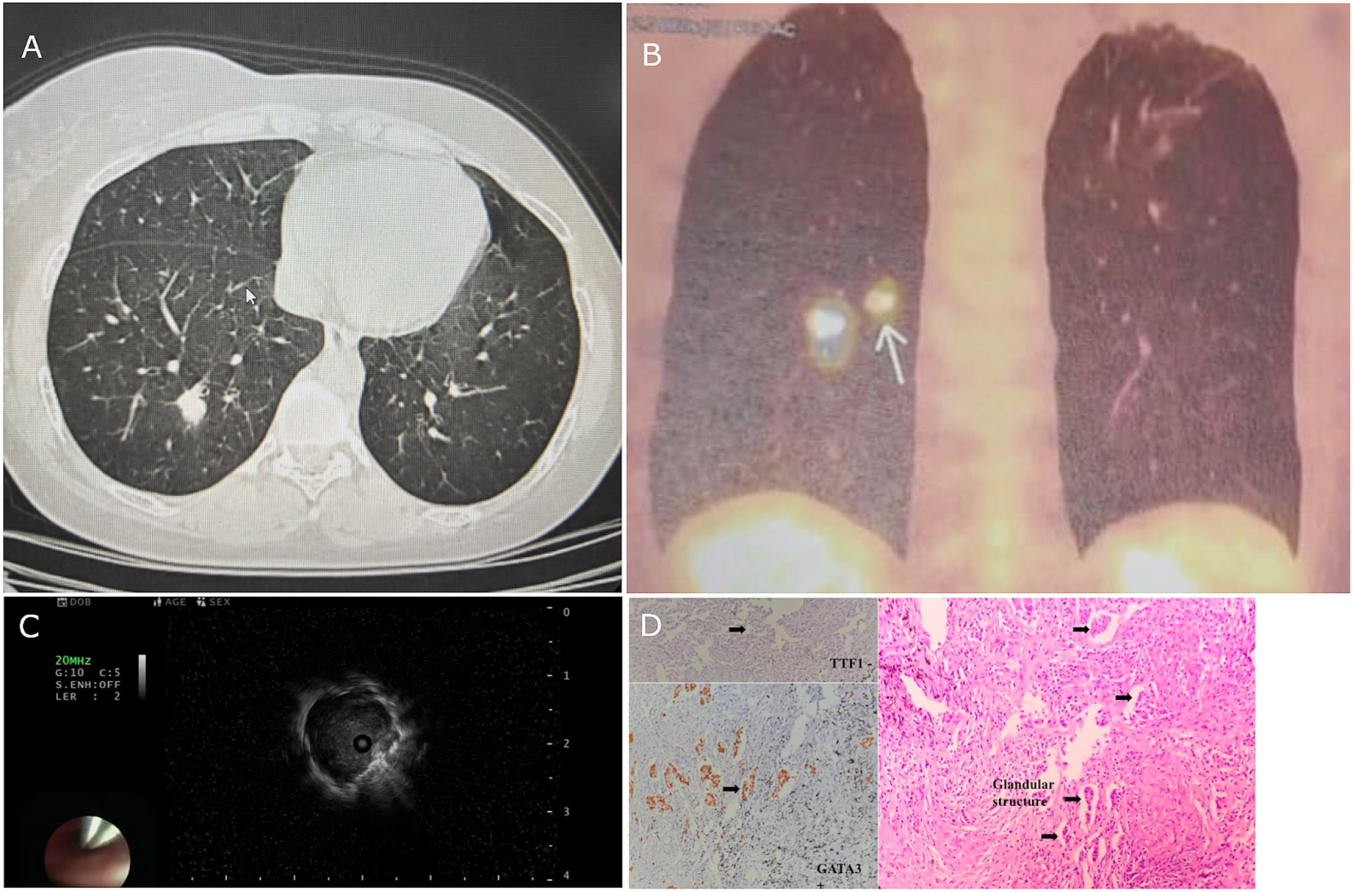
Radial EBUS‐guided transbronchial cryobiopsy: procedural workflow and histological diagnosis. (A) Axial chest CT scan showing a peripheral pulmonary lesion with a positive bronchus sign in the right upper lobe. (B) PET‐CT scan showing a focal hypermetabolic lesion (arrow) in the right lung, suggestive of malignancy. (C) Radial EBUS image confirming concentric positioning of the probe within the target lesion prior to sampling. (D) Histopathological section showing metastasis from breast carcinoma.

Among the 55 confirmed diagnoses, malignant etiology was the predominant finding. Primary lung cancer was identified in 38 patients, accounting for 52.1% of the total study population and 69.1% of the diagnosed cases. Metastatic disease to the lung was confirmed in six patients (8.2% of the total population) (Figure 1).

Benign etiologies were identified in a smaller subset of the cohort. Tuberculosis was diagnosed in six patients (8.2%), whereas nonspecific granulomatous inflammation was found in four patients (5.5%). One patient (1.4%) was diagnosed with organizing pneumonia. The distribution of histological subtypes and the overall diagnostic yield are detailed in Table 2.

**TABLE 2:** Diagnostic yield and etiology.

Diagnosis	Frequency (*N*)	% of total cohort
Diagnostic yield (overall)	55	75.3%
Malignant etiologies		
Primary lung cancer	38	52.1%
Metastasis	6	8.2%
Benign etiologies		
Tuberculosis	6	8.2%
Non necrosis granuloma	4	5.5%
Organizing pneumonia	1	1.4%
Non‐diagnostic	18	24.7%

### 3.3 Use of Virtual Navigation and Subgroup Analysis

The “Bronchus Archimède” virtual navigation system was used in 49 of the 73 patients (67.1%). In retrospective review, navigation was used in 100% of patients without a bronchus sign (15/15) and in 100% of patients with a lesion ≤ 20 mm (33/33); the remaining 10 navigated patients had lesions > 20 mm with a positive bronchus sign but were all located in the upper lobes, where the operator opted for navigation to facilitate access to anatomically challenging segments. No formal selection criteria were applied (see Methods). Results are presented in Table 3.

**TABLE 3:** Subgroup diagnostic yield with and without virtual navigation.

Stratum	Patients	Definitive diagnosis, *N* (%)
Overall cohort	73	55 (75.3%)
With virtual navigation	49	35 (71.4%)
Without virtual navigation	24	20 (83.3%)
Bronchus sign positive	58	44 (75.9%)
With virtual navigation	34	24 (70.6%)
Without virtual navigation	24	20 (83.3%)
Bronchus sign negative	15	11 (73.3%)
With virtual navigation	15	11 (73.3%)
Without virtual navigation	0	—
Lesions ≤ 20 mm	33	24 (72.7%)
With virtual navigation	33	24 (72.7%)
Without virtual navigation	0	—
Lesions > 20 mm	40	31 (77.5%)
With virtual navigation	16	11 (68.8%)
Without virtual navigation	24	20 (83.3%)

A post hoc subgroup analysis stratified by bronchus sign and by use of virtual navigation is presented in Table 3. Among the 58 patients with a positive bronchus sign, a definitive diagnosis was obtained in 44 (75.9%). Among the 15 patients without a bronchus sign, all of whom received virtual navigation, a definitive diagnosis was obtained in 11 (73.3%), comprising 7 primary lung cancers, 2 cases of tuberculosis, 1 metastasis, and 1 organizing pneumonia. The diagnostic yield was 35/49 (71.4%) when Archimède was used and 20/24 (83.3%) when it was not; this difference is consistent with a selection bias whereby virtual navigation was preferentially used in cases judged a priori more difficult (small, bronchus‐sign‐negative, or upper‐lobe lesions). Within the bronchus‐sign‐negative subgroup, one pyopneumothorax (the tuberculosis case discussed below) and two mild bleeding events were recorded; no severe bleeding occurred.

### 3.4 Safety and Complications

Safety outcomes were monitored rigorously, with a specific focus on pneumothorax and hemorrhagic complications. The incidence of pneumothorax and bleeding events is presented in Table 4.

**TABLE 4:** Safety and complications.

Complication	*N* (%)	Management/detail
Pneumothorax	6 (8.2%)	
Conservative management	2 (2.7%)	Observation only
Chest tube drainage	4 (5.5%)	Intervention required
Bleeding	14 (19.2%)	
Mild	14 (19.2%)	Balloon blocker/cold saline
Severe	0 (0.0%)	No transfusion/surgery required

#### 3.4.1 Pneumothorax

Pneumothorax was observed in six patients, representing an overall incidence rate of 8.2%. The management of these complications varied based on the clinical status of the patient and the volume of the pneumothorax. Conservative management, consisting of observation and supplemental oxygen without invasive intervention, was sufficient for two of the cases (33.3% of pneumothoraxes). Invasive management via chest tube drainage was required in four patients (66.7% of pneumothoraxes; 5.5% of the total study population).

A notable serious adverse event occurred in one patient diagnosed with tuberculosis. This patient developed a pyopneumothorax following the procedure, necessitating prolonged drainage and antibiotic therapy. This case highlights the potential risks associated with manipulating infectious cavitary lesions or necrotic tissue in high‐risk populations.

#### 3.4.2 Bleeding

Hemorrhagic complications were the most frequently recorded adverse event, with some degree of bleeding reported in 14 patients (19.2% of the total cohort). However, an analysis of bleeding severity indicates that all of these events were clinically insignificant, requiring the transient inflation of the prophylactic bronchial balloon blocker or the instillation of cold saline/vasoconstrictors.

Crucially, no cases of severe bleeding were observed in this study. There were no instances of hemodynamic instability, need for blood transfusion, or requirement for surgical intervention or admission to the intensive care unit for hemorrhage control.

## 4 Discussion

In this prospective observational study, we evaluated the diagnostic yield and safety profile of TBCB utilizing a 1.1‐mm cryoprobe guided by r‐EBUS for the diagnosis of PPLs. Our primary finding was a diagnostic yield of 75.3%, with a definitive histological diagnosis established in 55 of 73 patients. This yield was achieved with a standardized protocol involving general anesthesia and a prophylactic bronchial balloon blocker. Regarding safety, bleeding was recorded in 19.2% of cases, a rate consistent with the 18% reported by Herth et al. [7] and the 21.4% reported by Jiang et al. [9]. All bleeding events were mild and successfully managed with the balloon blocker; crucially, no cases of severe hemorrhage occurred. Pneumothorax occurred in 8.2% of cases, within the range reported in contemporary literature (6.6%–10%) [2, 7, 9]. These results suggest that the 1.1‐mm cryoprobe offers a favorable balance between diagnostic efficacy and safety, as recently corroborated by the randomized trial of Steinack et al. [10], particularly when integrated into a protocol that anticipates and proactively manages hemorrhagic risks.

The diagnostic yield of 75.3% observed in our cohort aligns closely with recent prospective data regarding the utility of 1.1‐mm cryoprobes. Steinack et al. recently reported a diagnostic yield of 72.4% using the same 1.1‐mm probe size in a randomized trial, finding no significant statistical difference between the 1.1‐mm and larger 1.7‐mm probes [10]. Similarly, Seong et al. reported a cryobiopsy‐specific yield of 74% in their “trimodality” study, which mirrors our findings almost exactly [11]. These comparisons are vital as they validate the efficacy of the thinner 1.1‐mm probe, which was selected for our study to enhance maneuverability in distal airways without sacrificing diagnostic power.

Our yield also compares favorably to studies utilizing larger cryoprobes. Jiang et al. achieved a 75% yield using a 1.9‐mm cryoprobe [9], and Gupta et al., in a systematic review, cited an aggregate yield of 74.2% for r‐EBUS‐guided cryobiopsy [1]. The fact that our 1.1‐mm probe achieved parity with these larger devices supports the hypothesis that probe flexibility and the ability to reach more peripheral generations of bronchi may compensate for the theoretically smaller freeze zone of a thinner probe.

The primary advantage of cryobiopsy over conventional forceps biopsy is the acquisition of larger, crush‐artifact‐free specimens. Although we did not perform a direct comparison with forceps in this study, the literature provides a robust mechanistic explanation for our diagnostic success. Kim et al. demonstrated that 1.1‐mm cryoprobes obtain tissue surface areas approximately five times larger than forceps (18.5 mm^2^ vs. 3.4 mm^2^) [12]. Similarly, Brown et al. found median sample sizes of 7.0 mm for cryobiopsy versus 2.5 mm for forceps [8].

This preservation of tissue architecture is clinically paramount. In our study, we successfully diagnosed lymphoma and granulomatous diseases (tuberculosis), pathologies that often require intact tissue architecture for definitive subtyping. Nogawa et al. highlighted this advantage in a case series of MALT lymphoma, where the 1.1‐mm probe achieved 100% yield due to architectural preservation [5]. Furthermore, adequate tissue volume is increasingly critical for molecular profiling in non–small cell lung cancer (NSCLC). Steinack et al. and Zarogoulidis et al. have both established that cryobiopsy specimens are superior for next‐generation sequencing (NGS) and PD‐L1 testing compared to forceps [5, 7]. Our ability to subtype 100% of the malignant cases in our cohort supports the notion that TBCB should be considered the standard of care when molecular analysis is anticipated.

A central point of discussion regarding TBCB is the risk of hemorrhage. Our study recorded bleeding in 19.2% of patients, a figure that is equivalent to the 18% reported by Herth et al. [7] or the 21.4% reported by Jiang et al. [9]. The prophylactic use of the bronchial balloon blocker allowed for immediate tamponade in cases of moderate bleeding, preventing escalation to severe hemorrhage.

More importantly, the severity profile in our study was excellent, with 0% severe bleeding and no requirement for transfusion or surgical intervention. This contrasts with Seong et al., who reported a 28% rate of Grade 3 bleeding despite similar diagnostic yields [11]. Our safety profile is more consistent with Steinack et al., who also reported zero severe bleeding events with the 1.1‐mm probe [10]. We attribute our avoidance of severe hemorrhage to the strict prophylactic use of a bronchial balloon blocker. By proactively occluding the lobar bronchus immediately after probe withdrawal, we ensured that even moderate bleeding (19.2% of our cohort) was contained and tamponaded before it could compromise the airway or hemodynamics. This finding supports the recommendation that balloon blockers should be mandatory during TBCB procedures [8]. The use of a small cryoprobe (1.1 mm) and a freeze time of 5 s may also have contributed to minimizing the risk of severe hemorrhage [8].

The incidence of pneumothorax in our study was 8.2%, which falls squarely within the range reported in contemporary literature. Taton et al. reported a 10% rate [2], Jiang et al. reported 7.1% [9], and Herth et al. reported 6.6% [7]. Although some studies utilizing ultrathin bronchoscopes have reported rates as low as 0%–1% [8, 10], our use of a standard 1.7‐mm working channel scope with a guide sheath likely reflects the “real‐world” risk profile of this procedure in a general interventional pulmonology setting.

A specific safety concern identified in our study was the occurrence of a pyopneumothorax in a patient with tuberculosis. This severe adverse event highlights the unique risks associated with cryobiopsy in necrotic or cavitary infectious lesions. Although cryobiopsy is effective for diagnosing TB (yield of 8.2% in our cohort), the freezing of fragile, infected tissue near the pleura carries a distinct risk of bronchopleural fistula formation. This aligns with warnings in the literature regarding the manipulation of cavitary lesions. Future protocols may need to consider excluding peripheral cavitary lesions from cryobiopsy or utilizing fluoroscopic guidance to ensure a greater margin from the pleura in suspected infectious cases.

Our study relied primarily on r‐EBUS for localization, with the “Bronchus Archimède” virtual navigation system used in 67.1% of cases at the operator's discretion. The presence of a CT bronchus sign was identified in 79.5% of our patients, a factor known to strongly predict diagnostic success. Benn et al. demonstrated that cryobiopsy yield drops significantly (to 60%) in lesions lacking a bronchus sign [6]. Conversely, Kho et al. described a “pinpoint” technique combining needle aspiration and cryobiopsy for extraluminal lesions, suggesting that advanced maneuvers can overcome the absence of a bronchus sign [13].

The subgroup analysis presented in Table 3 informs the contribution of virtual navigation to diagnostic performance. In the 15 bronchus‐sign‐negative patients, all of whom received virtual navigation, the diagnostic yield was 73.3% (11/15), only 2.6 percentage points below the 75.9% obtained in the bronchus‐sign‐positive group. This near‐equivalence is notable because, in the absence of any navigation support, the yield of cryobiopsy in bronchus‐sign‐negative lesions has been reported to drop to approximately 60% [6]. Our data therefore suggest that adjunctive laptop‐based virtual navigation substantially narrows the diagnostic gap and may compensate for the loss of bronchoscopic guidance that the bronchus sign normally provides. The unadjusted yield in cases performed with navigation (71.4%) was lower than in cases performed without it (83.3%), but this difference reflects the selection of inherently more difficult lesions (smaller, bronchus‐sign‐negative, or upper lobe) for navigation rather than any negative effect of the technology itself. From a practical standpoint, our results support the routine use of virtual navigation as an adjunct to r‐EBUS‐guided cryobiopsy whenever the lesion is small (≤ 20 mm), lacks a bronchus sign, or is located in an anatomically challenging segment, whereas r‐EBUS alone remains reasonable for larger, bronchus‐sign‐positive lesions in easily accessible territories. For the 24.7% of patients who were ultimately non‐diagnostic, more advanced navigation platforms (ENB, Robotics) or cone beam CT might offer additional incremental benefit; Graham et al. note that although these technologies reduce CT‐to‐body divergence, they come with significant costs [14]. Our data therefore support a stepwise, cost‐efficient strategy: r‐EBUS‐guided cryobiopsy with adjunctive laptop‐based virtual navigation as a first‐line approach, with more expensive navigational tools reserved for the residual subset of difficult cases that fail this initial strategy.

Our study has several limitations. First, it was a single‐center observational study without a randomized control arm comparing TBCB directly to forceps or needle aspiration. Although we can infer superiority based on historical controls and parallel literature [4, 7], a direct comparison within our specific population would have strengthened the conclusion. Second, no formal selection criteria for the use of virtual navigation were pre‐specified in the study protocol; navigation was assigned at the operator's discretion, with a strong preference for smaller, bronchus‐sign‐negative, or upper‐lobe lesions. This introduces a selection bias that limits the head‐to‐head comparison of yield with and without navigation, and the resulting subgroup analyses should be interpreted as exploratory rather than confirmatory; the moderate size of the bronchus‐sign‐negative subgroup (*n* = 15) further limits the precision of yield estimates within this stratum. Third, our definition of bleeding was not standardized to a specific scale (like the Nashville scale), which complicates direct statistical comparison with some registry studies. Finally, the sample size of 73 patients, although sufficient for a prospective observational cohort, limits our ability to perform robust subgroup analyses on smaller demographic clusters, such as specific lesion locations or sizes < 15 mm.

## 5 Conclusion

This prospective observational study demonstrates that TBCB utilizing a 1.1‐mm cryoprobe under radial EBUS guidance is a feasible and effective technique for diagnosing PPLs, achieving a diagnostic yield of 75.3%. Although minor bleeding events are frequent, the protocolized use of a prophylactic bronchial balloon blocker successfully prevented severe hemorrhagic complications, ensuring a favorable safety profile. These findings support the clinical utility of the 1.1‐mm cryoprobe as a valuable tool for obtaining high‐quality histological specimens, particularly when molecular profiling or subtyping is required. However, the incidence of pneumothorax necessitates continued vigilance, especially in patients with infectious cavitary disease. Future research should focus on large‐scale randomized controlled trials comparing this technique directly with emerging navigational platforms to further define its optimal role in the diagnostic algorithm for lung nodules.

## Author Contributions

In accordance with the ICMJE criteria, the individual contributions of the authors are as follows: Lamya Chrif Morand designed the study, performed research, collected data, analyzed data, and wrote the paper. Khalid Bouti analyzed data and revised the manuscript. Imane Saidi analyzed data and revised the manuscript. Soumia Fdil analyzed data and revised the manuscript. Anis Rafik analyzed data and revised the manuscript. All authors have participated sufficiently in the work to take public responsibility for the content and have read and approved the final version of the manuscript.

## Funding

The authors have nothing to report.

## Ethics Statement

The study was conducted in accordance with the Declaration of Helsinki and with Moroccan Law 28‐13 of September 17, 2015, on the protection of human subjects participating in biomedical research. Ethical approval was granted by the Hospital‐University Ethics Committee of Tangier (Comité d'Éthique Hospitalo‐Universitaire de Tanger, CEHUT), Faculty of Medicine and Pharmacy of Tangier, Abdelmalek Essaâdi University, Morocco, under reference number AC232MR/2026.

## Consent

Informed consent was obtained from all subjects involved in the study. Written informed consent has been obtained from the patients to publish this paper, including clinical images.

## Conflicts of Interest

The authors declare no conflicts of interest.

## Data Availability

The data presented in this study are available on request from the corresponding author.
